# Identification of host lncRNAs that impact Venezuelan equine encephalitis virus TC-83 replication

**DOI:** 10.1128/jvi.01353-25

**Published:** 2026-04-23

**Authors:** Mahgol Behnia, Chunyan Ye, Kim Somfleth, Olufunmilola M. Oyebamiji, Kathryn J. Brayer, Yan Guo, Scott A. Ness, Ram Savan, Steven B. Bradfute

**Affiliations:** 1 Center for Global Health, Department of Internal Medicine, University of New Mexico Health Sciences Center 12289, Albuquerque, New Mexico, USA; 2 Department of Immunology, University of Washington 7284, Seattle, Washington, USA; 3 Comprehensive Cancer Center, University of New Mexico 1104, Albuquerque, New Mexico, USA; Loyola University Chicago - Health Sciences Campus, Maywood, Illinois, USA

**Keywords:** snhg15, Venezuelan equine encephalitis virus, long non-coding RNA

## Abstract

Venezuelan equine encephalitis virus (VEEV) causes encephalitis in humans and equids, and there are no vaccines or therapeutics for humans. In recent years, non-coding RNAs have emerged as critical regulatory factors affecting different cellular pathways. Specifically, long non-coding RNAs (lncRNAs) have been identified as regulators of antiviral pathways; however, their role in VEEV infection has not been assessed. Here, we show differential expression of several lncRNAs in primary mouse target cells infected with a vaccine strain of VEEV (TC-83) but not a pathogenic strain (TrD). Among the differentially expressed genes (DEGs), suppressing lncRNA small nucleolar RNA host gene 15 (Snhg15) resulted in a 7-fold increase in TC-83 replication in primary mouse astrocytes. Knockdown of Snhg15 during TC-83 infection resulted in the suppression of ten genes, all of which were also increased during TC-83 infection along with Snhg15. Most of these genes are involved in antiviral responses. KEGG pathway analysis confirmed the suppression of both pattern recognition receptor and inflammatory pathways after Snhg15 knockdown. However, Snhg15 suppression did not significantly alter NF-kB signaling in TC-83-infected cells. These data are the first to identify lncRNA responses in encephalitic alphavirus infection and demonstrate important roles for these overlooked RNAs in VEEV infection.

**IMPORTANCE:**

Although many studies have reported differential expression of lncRNAs during viral infections, the lncRNA response to VEEV infection and its functional roles have not been previously characterized. In this study, we provide the first comprehensive analysis of host lncRNA expression in primary cells that are targeted during VEEV infection. We demonstrate that the expression of specific host lncRNAs is altered during VEEV infection and that modulation of these lncRNAs changes the expression of host antiviral and inflammatory pathways and impacts viral replication. These findings advance our understanding of VEEV-host interaction and shed light on previously unappreciated regulatory layers of infection. Given the absence of approved vaccines or antiviral therapies for VEEV, our work identifies novel host factors that may serve as potential targets for the development of anti-VEEV therapeutics upon further investigation.

## INTRODUCTION

Venezuelan equine encephalitis virus (VEEV) can cause encephalitis in humans and equines and has been responsible for large encephalitic outbreaks in North, Central, and South America (1). VEEV is a positive-strand RNA virus from the alphavirus genus in the family *Togaviridae*. In nature, both enzootic and epizootic strains of VEEV are transmitted through mosquito bites (2). However, VEEV is also highly infectious through aerosols and has previously been developed as a biological weapon (3, 4). These characteristics have led to the recognition of this virus as a potential biological terrorism agent, and it is classified as a Category B priority pathogen by the Centers for Disease Control and Prevention and the National Institutes of Health. VEEV infection leads to the sudden onset of flu-like symptoms 2–6 days after infection, including fever, fatigue, headache, myalgia, and nausea in humans (5). Highly lethal in equids, VEE disease is mostly self-limiting in humans, but in some cases, it causes encephalitis, resulting in a fatality rate of <1% in symptomatic patients. Despite the low fatality rate, 4%–14% of patients develop encephalitis, leading to permanent neurological sequelae (6). Extensive efforts to produce a VEEV vaccine resulted in VEEV TC-83, which was generated by attenuation of a virulent select agent VEEV strain, VEEV Trinidad Donkey (TrD) (7). However, VEEV TC-83 is only available to at-risk personnel and has questionable efficacy. The lack of a U.S. Food and Drug Administration (FDA)-approved vaccine or therapeutic for human use underscores the necessity of investigating the VEEV-host interaction to identify new therapeutic targets.

Previous studies have reported the induction of type I and II interferon (IFN-I and IFN-II) and proinflammatory mediators during VEEV infection in non-human primates, mouse models, and cell culture (8–13). However, other studies indicated that VEEV evades the host immune response by blocking expression of interferon-stimulated genes (ISGs), inducing host transcriptional shutoff, and causing host translational shutoff, which are mediated by VEEV capsid and nonstructural protein 2 (nsP2), respectively (14, 15). Despite the ability of the viruses to evade innate antiviral responses, most viral infections can be cleared by the hosts, suggesting the significance of additional host regulatory factors in the modulation of the host innate antiviral pathways.

Long noncoding RNAs (lncRNAs) are a group of regulatory RNAs that are longer than 200 nucleotides in length and lack protein-coding potential (16). Recent studies have identified lncRNAs as key regulators of signaling pathways that exert their regulatory effect through epigenetic, transcriptional, and post-transcriptional regulation of gene expression (17). lncRNAs regulate cellular processes like development, differentiation, and immune responses through the regulation of gene expression (18–20). Additionally, dysregulation of lncRNA expression is linked with different pathological conditions (21, 22).

Notably, a growing body of studies reports changes in cellular lncRNA expression during viral infections. These studies reveal that lncRNAs modulate innate antiviral responses using different mechanisms, including regulation of pathogen recognition receptor (PRR) activation, activation of transcription factors, and modulation of antiviral gene expression (23–25). Moreover, altering the expression of differentially expressed lncRNAs regulates viral infection. lncRNA LINC02574, whose expression is induced upon influenza A virus (IAV) infection, is a positive regulator of the innate antiviral response, as suppression of this lncRNA led to higher IAV replication in A549 cells (26). Conversely, siRNA-mediated disruption of lncRNA lncRHOXF1 in human trophectoderm progenitors during Sendai virus infection increased RIG-I and MDA-5 expression (27). These studies show the importance of lncRNAs in the modulation of viral infections, indicating that a deeper understanding of lncRNA function during viral infections may lead to the discovery of new cellular targets for treatment strategies.

In recent years, many studies have aimed to identify the function of lncRNAs during various viral infections. Despite this increasing effort, the lncRNA response to encephalitic alphaviruses and the regulatory impact of lncRNAs on their infection remains elusive. In this study, we investigated the lncRNA response to both wild-type (TrD) and vaccine strains (TC-83) of VEEV using mouse primary neurons and astrocytes, as VEEV-infected mice exhibit disease symptoms that closely resemble those observed in humans (8) and provide a valuable system for investigating the antiviral responses to VEEV infection and disease. Importantly, utilizing primary cells known to be targets of VEEV can provide valuable and more biologically relevant information about a viral infection and the subsequent cellular response.

Here, our RNA sequencing analysis showed significant alterations in lncRNA expression following TC-83 but not TrD infection in primary mouse astrocytes and neurons *in vitro*. Subsequent experiments demonstrated increased TC-83 replication and titer in response to the suppression of four lncRNAs. Further investigation of the lncRNA Small nucleolar host gene 15 (Snhg15) indicated a substantial decrease in the expression of antiviral genes after Snhg15 knockdown in primary mouse astrocytes infected with TC-83. Notably, expression of Irf1, Junb, Atf3, Relb, Pim1, Hbegf, Ccl5, Ankrd33b, and H2-K2 decreased in response to Snhg15 KD at all time points analyzed after infection. Furthermore, Gene Ontology and KEGG pathway analysis showed suppression of innate antiviral and inflammatory pathways in Snhg15 KD primary mouse astrocytes during VEEV TC-83 infection, with the NF-κB signaling pathway being suppressed at all time points post-infection. We investigated the impact of Snhg15 suppression on NF-kB signaling in the TC-83-infected cells using different methods. However, none of these assays showed a significant change in NF-kB signaling following Snhg15 suppression. Overall, our results suggest Snhg15 is a regulator of innate antiviral and inflammatory pathways during VEEV TC-83 infection.

## RESULTS

### RNA sequencing reveals differential lncRNA expression in response to VEEV TC-83 but not VEEV TrD infection in primary mouse astrocytes and neurons

**Fig 1:**
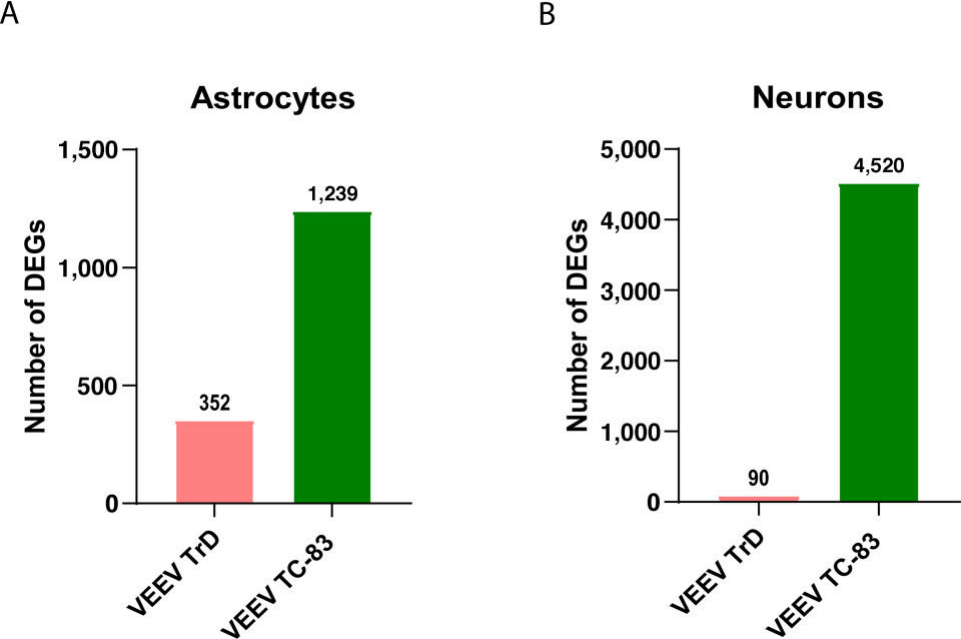
Cellular response to VEEV TrD vs. VEEV TC-83 in different cell types in the CNS. Comparison of the total number of differentially expressed mRNAs and lncRNAs in VEEV TrD- vs. VEEV TC-83-infected primary mouse astrocytes (**A**) and primary mouse neurons (**B**) at 24 h. The genes with p.adjust < 0.05 were identified as significantly modulated in RNA-seq analysis.

To explore the host lncRNA response to VEEV infection, we performed RNA sequencing in VEEV target cells infected with either wild-type (TrD) or live-attenuated (TC-83) VEEV strains and in uninfected controls. Since astrocytes and neurons are primary VEEV target cells *in vivo*, we focused our experiments on these cells. Primary mouse neurons and astrocytes were infected with either VEEV TrD or VEEV TC-83 at a multiplicity of infection (MOI) of 5 for 16 or 24 h or left uninfected as controls. Total RNA obtained from the infected and uninfected control cells was fragmented before processing, as previously described (28), to inactivate genomic viral RNA prior to RNA sequencing. RNA-seq analysis confirmed the successful infection of both mouse cell types and validated the replication of both VEEV strains in these cells (Fig. S1). TrD replicated more quickly than TC-83 in astrocytes, similar to previous findings in different cell types (Fig. S1C) (29). Analyses of differentially expressed mRNAs revealed a significant increase in the expression of genes involved in antiviral signaling pathways across both cell types and in response to both infections. However, the number of differentially expressed genes (DEGs) and the extent of antiviral pathway activation were different based on viral strain. In both primary mouse astrocytes and neurons, the cellular mRNA response was more robust in TC-83 infection, evidenced by a strikingly higher number of genes being modulated in TC-83-infected cells compared to TrD-infected cells (Fig. 1A and B). Differential gene expression analyses indicated differential expression of a greater number of antiviral genes in TC-83 vs. TrD-infected primary mouse astrocytes and neurons (Fig. S2). These results show a vastly different cellular mRNA response to infections in wild-type versus attenuated VEEV. Further KEGG pathway analysis revealed the activation of antiviral pathways in response to both strains of VEEV in primary mouse astrocytes and neurons (Fig. S3 and S4). However, the number of enriched antiviral pathways was higher in TC-83 infection of these cells. These results suggest that the wild-type strain of VEEV efficiently evades the cellular antiviral responses in the central nervous system.

**Fig 2:**
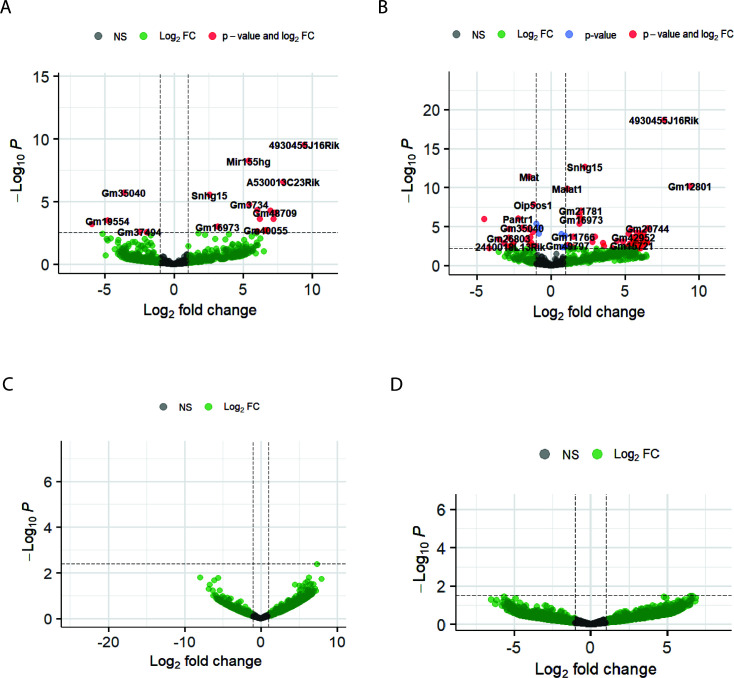
The host cellular lncRNA response to VEEV strains at 24 hpi. (**A and B**) Volcano plots show DE-lncRNAs in primary mouse astrocytes (**A**) and primary mouse neurons (**B**) infected with VEEV TC-83. (**C and D**) Volcano plots show DE-lncRNAs in primary mouse astrocytes (**C**) and primary mouse neurons (**D**) infected with VEEV TrD. All the plots show the results from RNA-seq at 24 hpi. The *P*-value threshold is adjusted to show p.adj value = 0.05.

Notably, we observed a significant difference in the host lncRNA response to these infections. TC-83 infection led to substantial changes in the expression of cellular lncRNAs in primary mouse astrocytes and neurons. Particularly, 8 and 24 lncRNAs in primary mouse astrocytes and 22 and 78 lncRNAs in primary mouse neurons were differentially expressed at 16 and 24 hpi, respectively (Fig. S5A and B; Fig. 2A and B). In contrast, TrD infection did not affect the expression of host lncRNAs in either cell type at these time points (Fig. S5C and D; Fig. 2C and D).

Comparing the list of significantly altered lncRNAs in TC-83-infected primary mouse astrocytes and neurons revealed the modulation of nine lncRNAs shared in both of these cell types, with eight showing a consistent trend in both cell types at 24 hpi. Interestingly, only two of these shared lncRNAs, small nucleolar RNA host gene 15 (Snhg15) and myocardial infarction-associated transcript (Miat), have been previously characterized, while the function of the remaining lncRNAs is still unknown. These results demonstrate a distinct host lncRNA response to VEEV TC-83 infection in the cells of the central nervous system.

The vaccine strains of viruses elicit robust but safe immune responses to the viral infection. The robust mRNA and lncRNA responses to TC-83 infection but not pathogenic TrD observed in our study suggest activation of the pathways that are typically controlled or suppressed by wild-type VEEV. Given that lncRNAs can play key roles in regulating antiviral pathways, modulation of lncRNAs in response to TC-83 infection, but not TrD infection, suggests that these lncRNAs may serve as key regulators of host antiviral signaling pathways by modulating the viral infection.

### lncRNAs modulated in VEEV TC-83-infected primary mouse astrocytes regulate viral infection

To identify lncRNAs with anti-VEEV activity, we selected a subset of eight lncRNAs identified as differentially expressed in our RNA-seq analysis (Table 1). Among these, three lncRNAs, including Snhg15, Miat, and 4930455J16Rik, were significantly modulated in both TC-83-infected primary mouse astrocytes and neurons. The remaining five lncRNAs were differentially expressed only during TC-83 infection in primary mouse astrocytes. Although both astrocytes and neurons are vital VEEV target cells *in vivo*, because astrocytes are a place for VEEV replication and contribute to brain inflammation, we used these cells to investigate the potential regulatory function of modulated lncRNAs during TC-83 infection.

**TABLE 1:** Differentially expressed lncRNAs selected for RNAi screening

lncRNA	Species	*P*-adjusted value^*a*^	Log2 fold change^*a*^
4930455J16Rik	Mouse	9.35E-08	9.427488
Mir155hg	Human/mouse	9.22E-07	5.391059
A530013C23Rik	Mouse	2.84E-05	7.881687
Snhg15	Human/mouse	0.0002	2.561977
Gm3734	Mouse	0.0009	5.455434
Miat	Human/mouse	0.02	−5.92181
3110039M20Rik	Mouse	0.04	6.035248
Mir9-3hg	Human/mouse	0.05	−1.96377

^*a*^ *P*-adj and log2FC values at 24 hpi.

**Fig 3:**
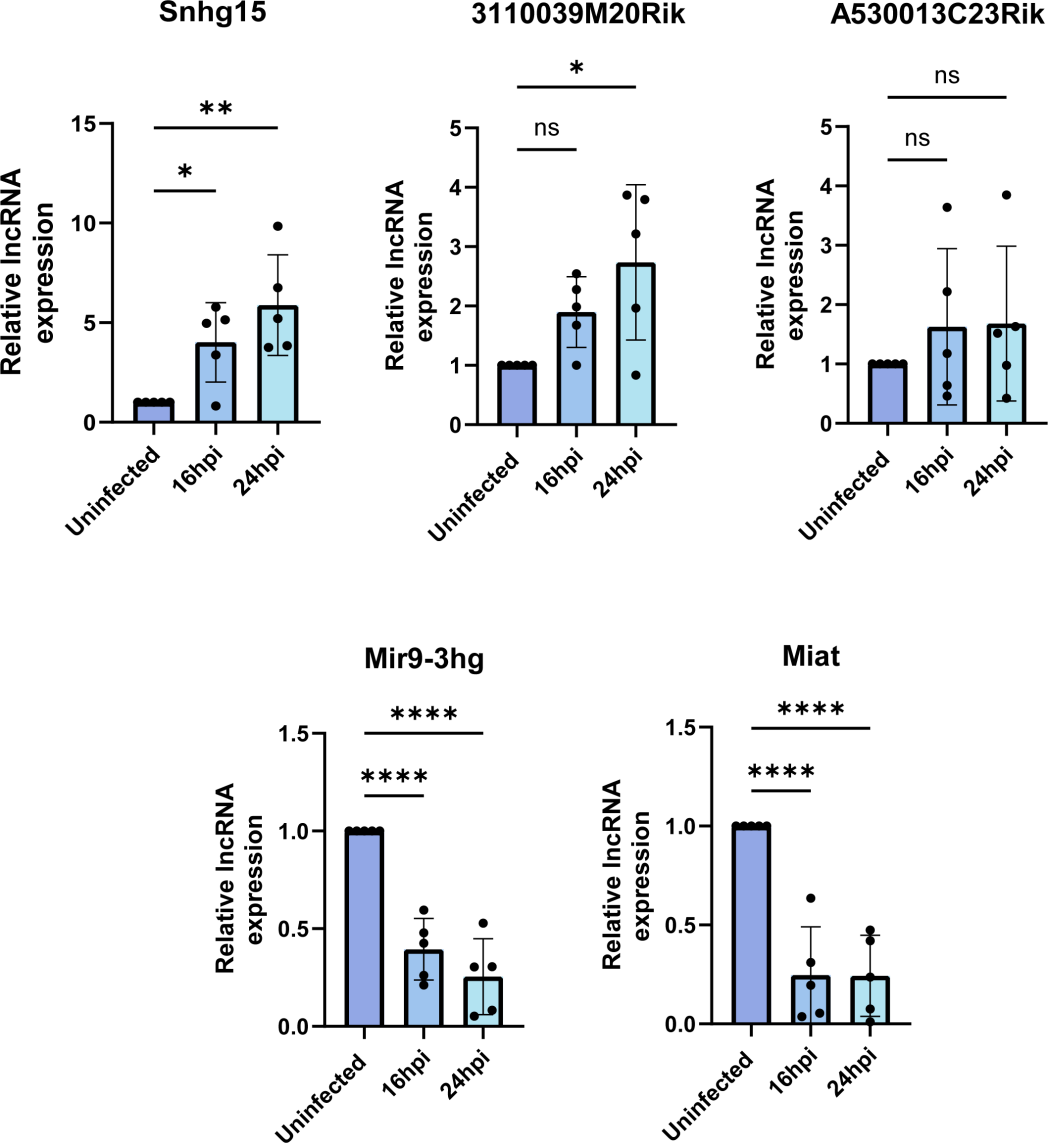
Validation of lncRNA expression using RT-qPCR. Expression changes in five lncRNAs were successfully validated in VEEV TC83-infected (MOI 5) primary mouse astrocytes at 16 and 24 hpi using TaqMan assays. The expression of each lncRNA was measured relative to GAPDH and normalized to the lncRNA expression level in the uninfected control. Each dot represents a biological replicate. One-way ANOVA followed by multiple comparisons was used to measure the statistical significance. ns: adj. *P*-value > 0.05; * adj. *P*-value < 0.05; ** adj. *P*-value < 0.01; **** adj. *P*-value < 0.0001.

To investigate the potential regulation of VEEV infection by differentially expressed lncRNAs, we began by validating the expression change in the selected lncRNAs during TC-83 infection using RT-qPCR. To this end, we infected primary mouse astrocytes with TC-83 (MOI 5) or left them uninfected as controls. The cells were lysed at 16 and 24 hpi, and total RNA extracted from them was subjected to TaqMan assays targeting the lncRNA of interest and GAPDH as an internal control. These validations were performed in five biological replicates. Among the eight selected lncRNAs, Snhg15, Mir9-3hg, Miat, A530013C23Rik, and 3110039M20Rik showed expression changes consistent with our RNA-seq results in at least three biological replicates at both time points post-infection (Fig. 3).

**Fig 4:**
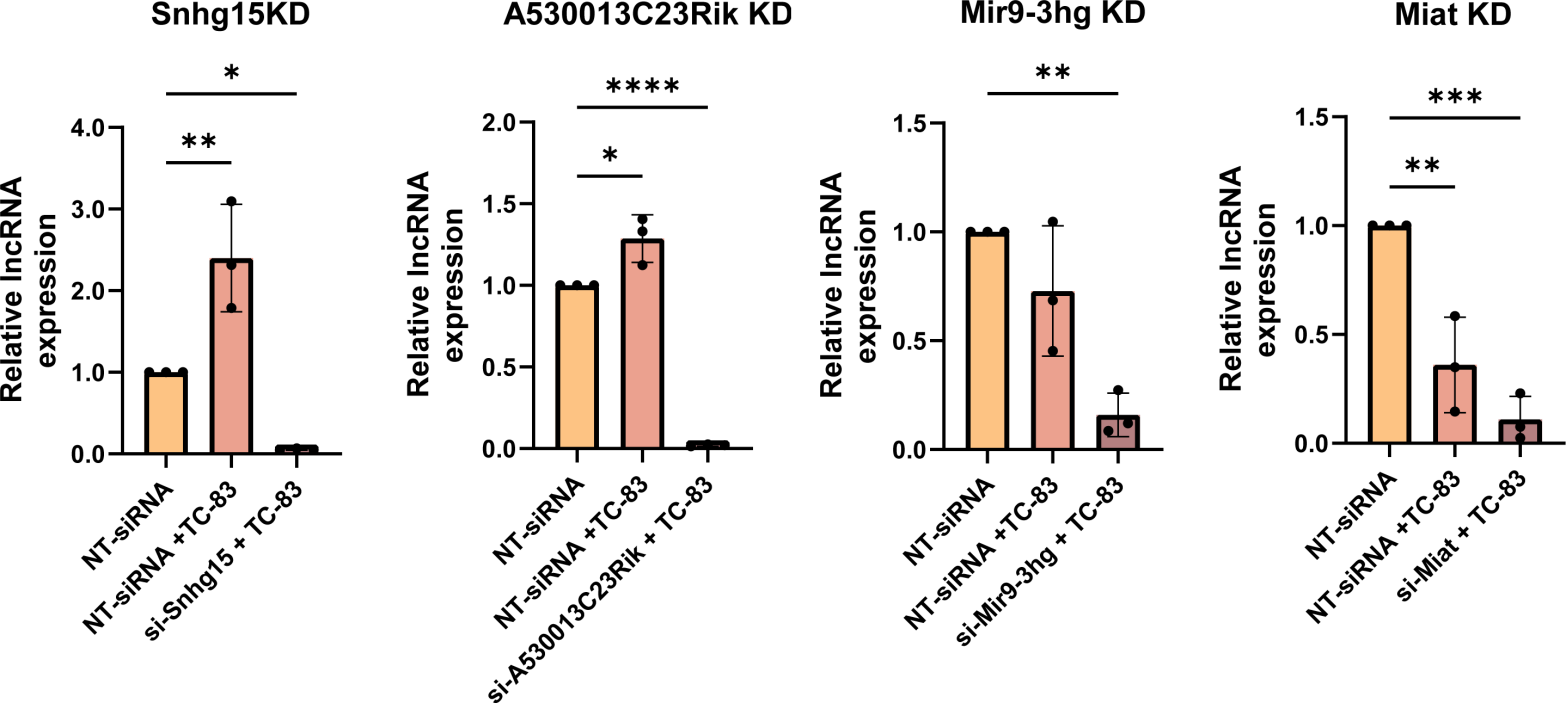
Four lncRNAs were suppressed using siRNAs in TC-83-infected primary mouse astrocytes. lncRNA suppression using siRNA was validated using RT-qPCR. The cells were transfected with either NT-siRNAs or si-lncRNA, followed by infection with TC-83 at an MOI of 5 for A530013C23Rik KD, Mir9-3hg KD, and Miat KD, and an MOI of 0.5 for Snhg15 KD cells. lncRNA expressions were measured using TaqMan assays at 24 hpi. The lncRNA expression was calculated relative to GAPDH and normalized to the lncRNA expression level in uninfected cells. Each symbol represents a biological replicate (*n* = 3). The statistical significance of lncRNA expression change was tested using one-way ANOVA with multiple comparisons. ns: adj. *P*-value > 0.05; * adj. *P*-value < 0.05; ** adj. *P*-value < 0.01; **** adj. *P*-value < 0.0001.

Next, we performed an RNAi screen to investigate the potential regulatory impact of these validated lncRNAs on TC-83 replication and titer. For this purpose, we first confirmed lncRNA suppression using RT-qPCR. To this end, we transfected primary mouse astrocytes with siRNAs targeting the lncRNA of interest and then infected them with TC-83 (MOI 5). The lncRNA expression in this group was compared to cells transfected with non-targeting siRNA (to measure basal lncRNA expression) and cells transfected with non-targeting siRNA followed by TC-83 infection (to measure lncRNA expression changes during infection). Among the five validated lncRNAs, expressions of four (Snhg15, A530013C23Rik, Mir9-3hg, and Miat) were successfully suppressed using siRNAs in TC-83-infected primary mouse astrocytes (Fig. 4).

**Fig 5:**
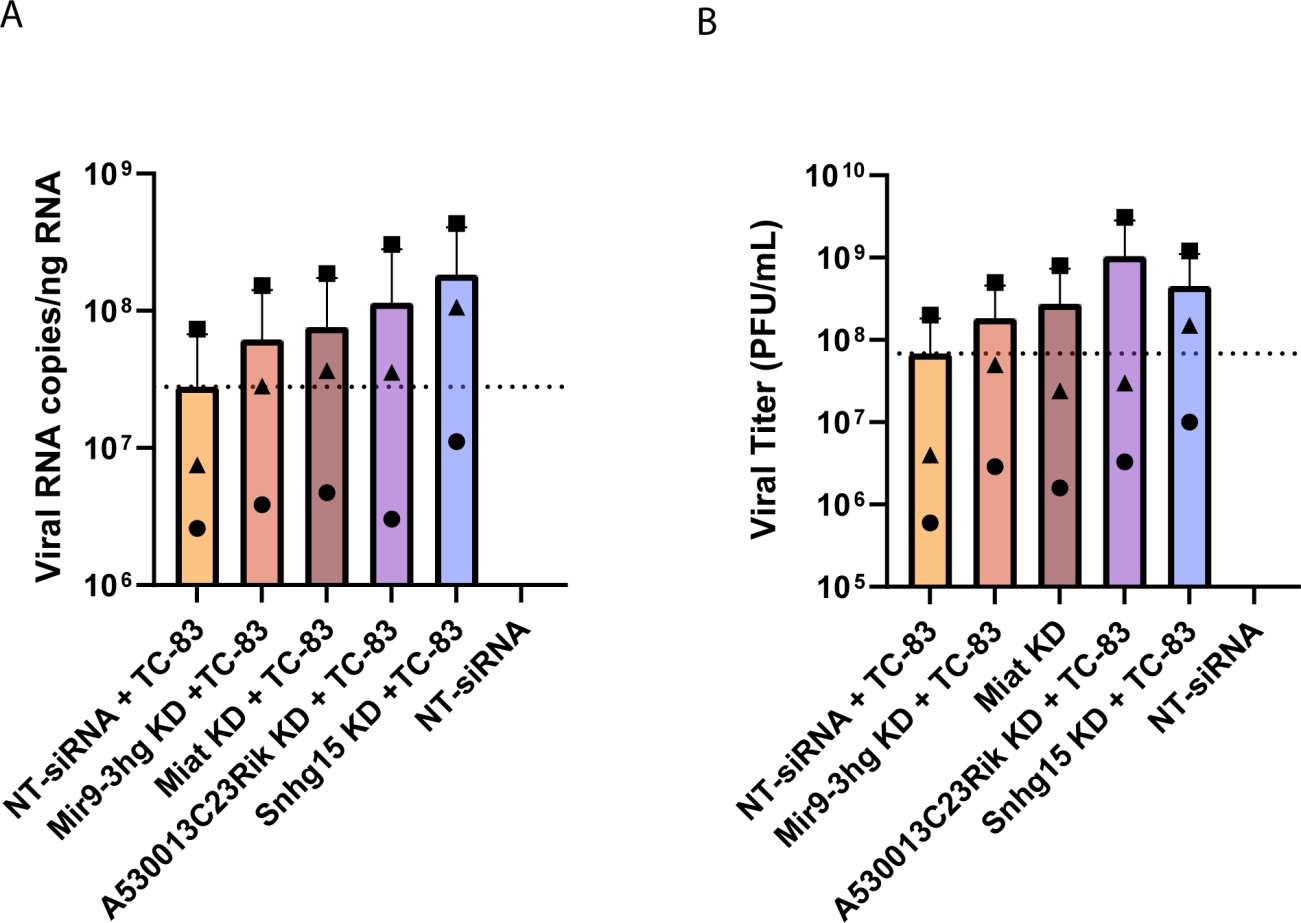
Suppression of three lncRNAs changed VEEV TC-83 replication and titer. (**A**) The effect of lncRNA KD on VEEV TC-83 RNA copy number was measured using a TaqMan assay targeting the VEEV capsid. Capsid copy numbers were measured using a standard curve generated based on the VEEV TC-83 plasmid and divided by the nanogram of RNA used in each reaction. (**B**) lncRNA suppression increased VEEV titer. Supernatants from lncRNA-suppressed cells infected with VEEV TC-83 (MOI 5) for 24 h were used to perform plaque assays. Plaques were counted at 48 hpi, and PFU/mL was calculated for all samples. Shapes correspond to the same biological sample across conditions.

To evaluate the regulatory impact of these four lncRNAs on VEEV replication, we performed TaqMan assays targeting VEEV capsid RNA in lncRNA-suppressed cells and controls. Primary mouse astrocytes transfected with a pool of four non-targeting siRNA infected with VEEV TC-83 (MOI 5) for 24 h served as a control for viral replication. Changes in the number of VEEV TC-83 genomes were quantified by generating a standard curve using a plasmid containing the complete VEEV TC-83 sequence in our RT-qPCR assays. Our results showed that the suppression of all four lncRNAs increased VEEV TC-83 RNA copy number, with Snhg15 knockdown leading to a 7-fold increase (Fig. 5A). To further investigate the effect of lncRNA suppression on VEEV titer, we collected the supernatants from lncRNA knockdown and control cells and performed plaque assay. Consistent with the RT-qPCR results, suppression of all four lncRNAs increased VEEV TC-83 titer, with lncRNAs A530013C23Rik and Snhg15 suppression resulting in the highest increase (Fig. 5B). These findings suggest the selected lncRNAs are regulators of VEEV replication. Among the four lncRNAs studied, suppression of Snhg15 had a pronounced effect on both viral RNA copy number and the number of infectious virions. Therefore, we selected Snhg15 for further functional analysis during VEEV infection.

### lncRNA Snhg15 may regulate VEEV TC-83 replication by modulating antiviral response to infection

After validating the effect of Snhg15 suppression on VEEV TC-83 replication and titer, we next measured the impact of Snhg15 knockdown on antiviral gene expression during TC-83 infection. Using RNA sequencing, we analyzed gene expression in primary mouse astrocytes with and without Snhg15 suppression at 8, 16, and 24 h after TC-83 infection. Control cells were transfected with a pool of four non-targeting siRNAs before infection with TC-83 to account for any transfection and infection effects on gene expression.

**Fig 6:**
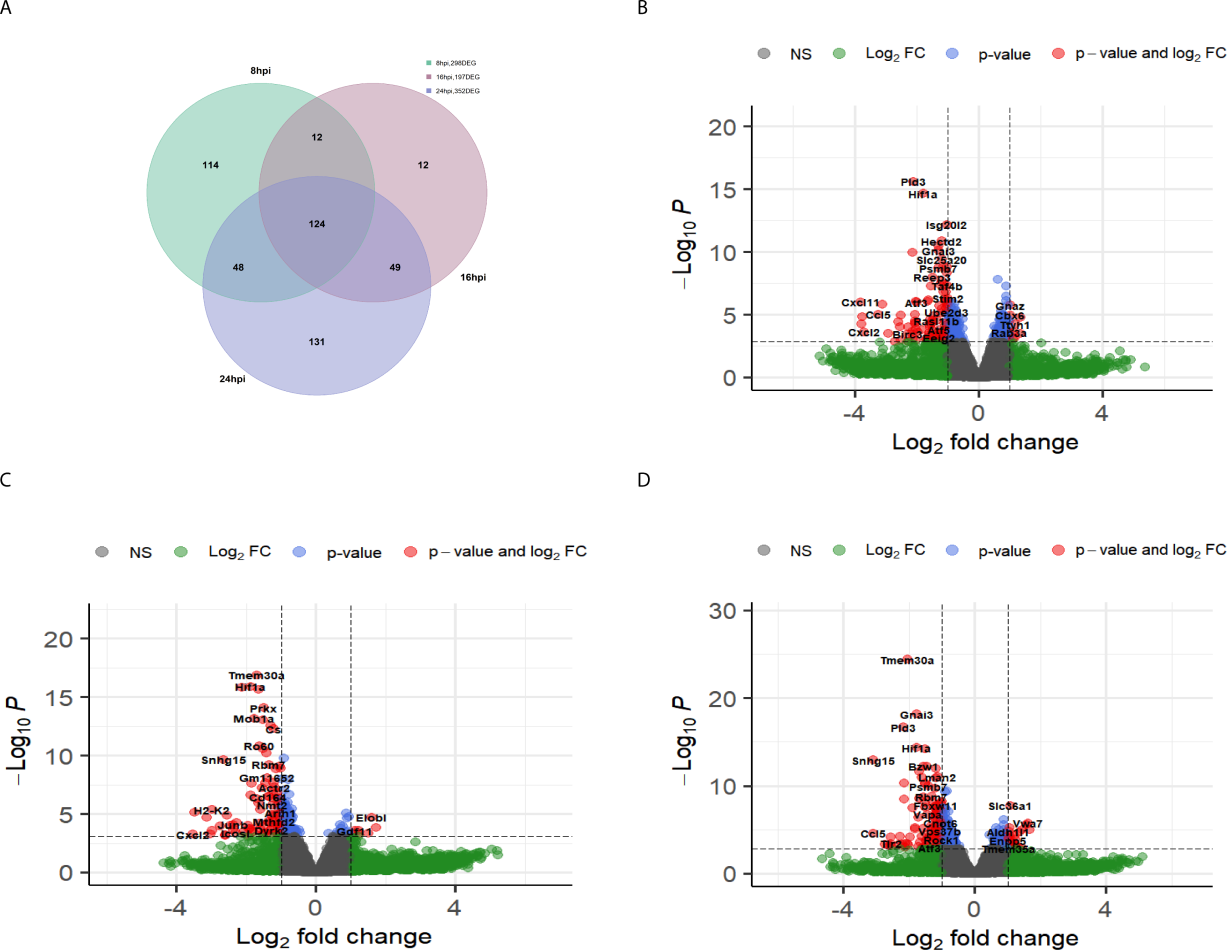
Snhg15 suppression decreased the expression of genes involved in the inflammatory response to viral infection. (**A**) The Venn diagram shows the number of differentially expressed genes in Snhg15-suppressed and NT-siRNA-treated primary mouse astrocytes, at 8, 16, and 24 hpi with VEEV TC-83 (MOI 5). (**B–D**) Volcano plots of differentially expressed genes in Snhg15KD vs. non-targeting siRNA-treated primary mouse astrocytes at different time points post-infection with VEEV TC-83 (MOI 5) at 8 hpi (B), 16 hpi (C), and 24 hpi (D).

Our differential gene expression analysis revealed significant alterations (*P*adj < 0.05, log2FC > 1) in the expression of 298, 197, and 352 host genes between Snhg15KD and control cells at 8, 16, and 24 h post-infection, respectively. Notably, the expression of 124 genes was changed across all time points after TC-83 infection in Snhg15KD cells (Fig. 6A). Interestingly, many of these genes encoded proteins involved in antiviral signaling pathways, suggesting Snhg15 as a regulator of antiviral pathways (Fig. 6B through D). To identify genes directly affected by Snhg15 expression, we compared the expression changes in these 124 genes with their expression changes observed during TC-83 infection, which was obtained from our primary RNA-seq data. This analysis revealed that a subset of 10 genes, including activating transcription factor 3 (Atf3), interferon regulatory factor 1 (Irf1), Junb, Relb, Pim1, histocompatibility 2, K region locus 2 (H2-K2), C-C motif chemokine ligand 5 (Ccl5), heparin-binding EGF-like growth factor like (Hbegf), NFKB inhibitor epsilon (Nfkbie), and ankyrin repeat domain 33B (Ankrd33b), exhibited a significant increase in expression during TC-83 infection, coinciding with increased Snhg15 expression. Importantly, the expression of these genes was suppressed during Snhg15 KD in the presence of TC-83 infection. Notably, most of these genes are involved in cellular antiviral responses (Table 2). For example, Irf1, Relb, and Atf3 are transcription factors that have been previously studied for their roles in regulating antiviral responses (30–32).

**TABLE 2:** Genes whose expression positively correlated with Snhg15 expression and their functions

Gene	Function
Irf1	Regulation of antiviral and inflammatory responses through the induction of IFNβ, ISGs, TLR2, and TLR3
Nfkbie	Regulation of inflammatory response through the inhibition of the NF-kB signaling pathway
Relb	NF-kB transcription factor involved in the regulation of NF-kB-induced genes
Ccl5	Chemokine involved in the recruitment of leukocytes to the site of inflammation.
Junb	AP1 transcription factor involved in the cellular response to stress
Atf3	Stress-induced transcription factor involved in the regulation of antiviral gene expression, e.g., STAT1
Pim1	Serine-threonine kinase involved in the suppression of interferon signaling and ISG production
Hbegf	Involved in influenza A entry
Ankrd33b	Function unknown
H2-K2	Function unknown

Additionally, decreased transcription of Tlr2, a pattern recognition receptor involved in the innate antiviral response, was observed in Snhg15KD cells at 16 and 24 hpi. The chemokines Cxcl1 and Cxcl2, which act as a chemoattractant for various immune cells during viral infection, were substantially decreased at 8 and 16 h post-infection in Snhg15-suppressed cells. We investigated our RNA-seq data from TC-83-infected primary mouse astrocytes to examine the expression levels of Tlr2, Cxcl1, and Cxcl2. We observed that these genes were upregulated during infection, coinciding with the increased expression of Snhg15 in response to TC-83 infection. This observation might suggest a more direct Snhg15 regulatory impact on these genes. Overall, the suppression of genes involved in antiviral responses, along with increased TC-83 replication and titer observed in Snhg15KD cells, suggests that Snhg15 is a critical regulator of the antiviral response to VEEV infection.

To further investigate the potential regulatory impact of Snhg15 on the antiviral cellular response during VEEV infection, we performed KEGG pathway analyses. As expected, these analyses revealed the downregulation of several antiviral pathways in Snhg15 KD cells during TC-83 infection. Notably, key innate antiviral pathways, such as the JAK-STAT signaling pathway, which is responsible for the production of type I interferons and their downstream signaling, were suppressed in Snhg15 KD cells at earlier time points of infection. This finding coincided with a non-statistically significant decrease in the transcript level of interferon β (IFN-β), which was observed in Snhg15 KD cells (6-fold decrease at 8 hpi, 3.8-fold at 16 hpi, and 2.7-fold at 24 hpi). Additionally, a comparison of all enriched pathways in Snhg15 KD cells at different time points post-infection showed the suppression of pathways involved in viral pathogen recognition, such as TLR, RIG-I-like receptor, NOD-like receptor, and C-type lectin receptor signaling pathways. These findings suggest that Snhg15 serves as a positive regulator of the cellular antiviral responses during VEEV infection.

**Fig 7:**
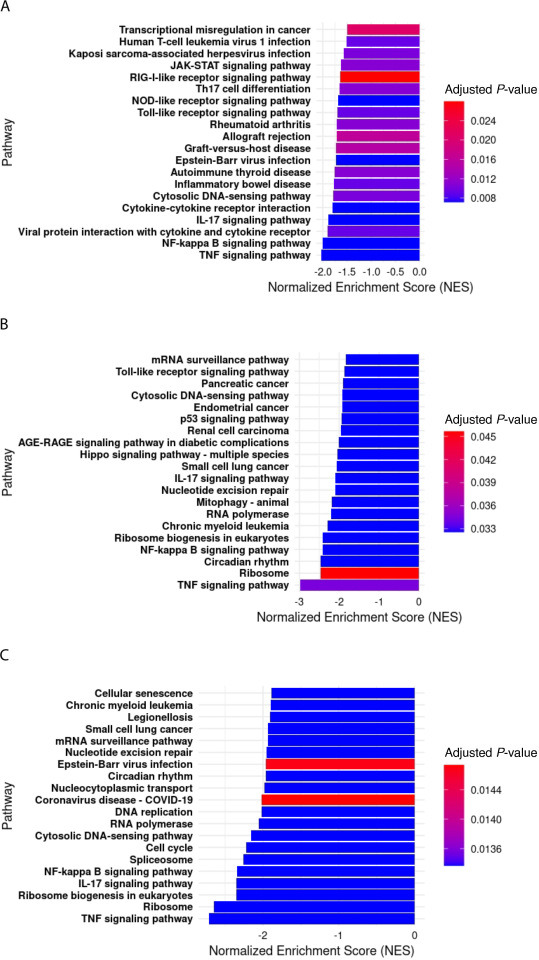
Snhg15 suppression of antiviral pathways. (**A-C**) Bar plots of the top 20 negatively enriched KEGG pathways in Snhg15KD primary mouse astrocytes at 8 hpi (**A**), 16 hpi (**B**), and 24 hpi (**C**) with VEEV TC-83 (MOI 5). The *P*.adj cutoff value for the KEGG pathway analysis is <0.05.

Moreover, Snhg15 suppression was associated with downregulation of inflammatory pathways, including NF-kB, TNF, and IL-17 signaling, all of which are involved in the host antiviral response. Looking more deeply into KEGG pathway analysis showed that NF-kB, TNF, and IL-17 signaling pathways were among the top 10 downregulated pathways at all analyzed time points post-infection in Snhg15-suppressed cells (Fig. 7A through C). Since the regulation of the NF-kB signaling pathway has been previously reported by both Snhg15 and VEEV (33, 34), we tested the impact of Snhg15 KD on NF-kB signaling during the VEEV infection.

### Snhg15 knockdown does not affect NF-kB signaling in primary mouse astrocytes

We tested the impact of Snhg15 suppression on NF-kB signaling using immunofluorescent microscopy, western blot, and ELISA. For immunofluorescent microscopy, we assessed the changes in p65 nuclear localization in response to Snhg15 suppression in TC-83 infected cells compared to controls, which were cells transfected with a pool of non-targeting siRNAs followed by infection with TC-83 (MOI 5). The cells transfected with a pool of non-targeting siRNAs and left uninfected were used as the control for the transfection effect. Our results from immunofluorescent microscopy showed a non-significant decrease in p65 nuclear localization in Snhg15 KD cells after TC-83 infection (Fig. S6).

We further investigated the impact of Snhg15 suppression on NF-kB transcription factor activation by measuring changes in p65 and phosphorylated p65 levels in Snhg15 KD cells. p65 is the most predominant transcription factor in the NF-kB transcription factor family. The phosphorylation of p65 is the active form of p65 that is found in the nucleus. Nuclear localization of p65 leads to transcription of its target genes, including proinflammatory genes (35). Our western blot results did not show alteration of p65 protein level or the phosphorylation level of this protein in Snhg15 KD primary mouse astrocytes, suggesting that the NF-kB pathway was neither activated nor suppressed in response to Snhg15 knockdown (Fig. S7A).

While none of our previous assays showed changes in NF-kB signaling in response to Snhg15 suppression, we further investigated this effect by performing ELISA assays to measure the potential changes in MIP-2 (CXCL2), KC (CXCL1), and RANTES (CCL5) and IL6 protein levels in the supernatant of Snhg15-suppressed TC-83-infected cells. These proteins are known to be induced by NF-kB signaling (36–38). The ELISA assay results showed no statistical change in the CCL5, CXCL2, CXCL1, or IL6 expression levels in response to Snhg15 suppression (Fig. S7). Altogether, these results show that Snhg15 is not the major regulator of the NF-kB signaling pathway. Further investigations are needed to determine this lncRNA’s function during VEEV infection.

## DISCUSSION

Modulation of cellular antiviral responses by lncRNAs has been reported during some viral infections (39). However, the lncRNA response to encephalitic alphaviral infection has not been reported. Here, for the first time, we report the lncRNA response to VEEV infection and introduce the regulatory impact of lncRNAs on this viral infection. Our RNA-seq analysis showed significant modulation of several lncRNAs in response to TC-83 infection in primary mouse astrocytes and neurons. However, this modulation was not observed in TrD infection of these cells (Fig. S5 ; Fig. 2). Suppression of both mRNA and lncRNA responses in TrD compared to TC-83 infection correlated with more rapid TrD replication in astrocytes, suggesting that suppression of cellular RNA responses to infection correlates with increased VEEV replication (Fig. S1). This has been shown in other cell types previously (29). This could be due to antiviral mRNAs and lncRNAs being suppressed in TrD but not in TC-83 infection.

The observed differences in lncRNA responses to various strains of VEEV in astrocytes and neurons may be due to differences in cellular antiviral responses to these infections. We identified 1,088 protein-coding genes whose expression specifically changed in response to TC-83 infection in primary mouse astrocytes at 24 hpi, many of which are essential for antiviral responses. In contrast, only a few genes involved in antiviral responses were upregulated in TrD-infected astrocytes, all of which were also increased during TC-83 infection. These findings are in contrast with previous reports indicating a more robust antiviral response to neurovirulent VEEV (VEEV 3000) compared to partially neurovirulent VEEV (VEEV 3034) infection in mice (40). The difference between our results and the results from Gupta et al. could be due to the different attenuated viruses used in these experiments. While the VEEV 3034 genome contains only one point mutation at E1 position 272, TC-83 differs from the wild-type strain by 11 point mutations (8). Wild-type VEEV evades the host immune response by inducing host transcriptional and translational shutoff (14, 15, 41). Previous studies (41) have shown that unlike TrD, TC-83 is unable to inhibit TNF-α-induced upregulation of NF-kB signaling activation and IFN-β-induced activation of ISRE and STAT-1 reporters, all of which are due to a mutation in the nucleotide at position 3 in the 5′ UTR of the viral genome. Therefore, the increased expression of antiviral genes and activation of additional antiviral pathways in response to TC-83 might be due to the activation of pathways that are potentially evaded by TrD in astrocytes. KEGG pathway analyses confirmed the activation of a greater number of antiviral signaling pathways in TC-83-infected astrocytes and neurons compared to TrD-infected cells, some of which were exclusively enriched during infection with the vaccine strain (Fig. S3 and S4). Particularly, several innate antiviral pathways, including RIG-I-like receptor signaling, C-type lectin receptor signaling, and MAPK signaling pathways, were positively enriched in TC-83 infection of astrocytes, but not in TrD infection of these cells (Fig. S3). Similarly, the JAK-STAT signaling pathway, the NOD-like receptor signaling pathway, and the NF-kB receptor signaling pathway were identified as positively enriched in TC-83-infected, but not TrD-infected, primary mouse neurons (Fig. S4).

We demonstrated that suppression of four lncRNAs, including Snhg15, Mir9-3hg, A530013C23Rik, and Miat, regulates VEEV TC-83 viral titers, as measured by capsid RNA levels in cells as well as plaque-forming virus in supernatants (Fig. 5). Using RNA-seq and differential gene expression analysis, we identified a subset of 10 genes (Atf3, Irf1, Junb, Relb, Nfkbie, Ccl5, Pim1, H2-K2, Hbegf, and Ankrd33b) whose expression was positively correlated with Snhg15 expression in Snhg15 KD and normal Snhg15 expressing TC-83-infected primary mouse astrocytes at all time points after infection (Table 3). Most of these genes are known regulators of antiviral and inflammatory responses (30–32, 42, 43), suggesting that Snhg15 is a regulator of antiviral and inflammatory responses during VEEV TC-83 infection.

The decreased expression of Relb, Nfkbie, Cxcl1, Cxcl2, Ccl5, and Pim1 suggests decreased enrichment of the NF-kB signaling pathway, as these genes are either products or modulators of this signaling pathway (37, 44–47). Specifically, the expression of Nfkbie (an inhibitor of NF-kB) and Relb (a Nf-kB transcription factor), the key components of the NF-kB signaling pathway, decreased across all time points post-infection in Snhg15 KD cells. Furthermore, Snhg15 suppression led to decreased expression of Cxcl1 and Cxcl2 at 8 and 16 hpi, as well as Ccl5 at all time points in TC-83-infected primary mouse astrocytes. Notably, the Relb, Cxcl1, Cxcl2, and Ccl5 expressions are induced by NF-kB transcription factors (37, 46, 48). Our KEGG pathway analysis confirmed negative enrichment of the NF-kB signaling pathway at all time points tested post-infection in Snhg15 KD cells and placed it among the top 10 suppressed pathways (Fig. 7A through C). Moreover, previous reports showed Snhg15 as both a positive and a negative regulator of the NF-kB signaling pathway in different disease conditions (33, 34). Therefore, we hypothesized that Snhg15 regulates VEEV TC-83 infection by modulating NF-kB signaling, which, in turn, controls proinflammatory cytokine and chemokine production.

We conducted multiple assays to determine if Snhg15 modulates the NF-kB pathway. We first investigated the impact of Snhg15 suppression on NF-kB activation by assessing p65 nuclear localization, p65 phosphorylation, and protein expression of downstream NF-kB genes. None of these assays revealed the role of NF-kB in Snhg15 mechanism of action after TC-83 infection, suggesting the use of other cellular pathways.

Snhg15 suppression decreased the expression of interferon regulatory factor 1 (Irf1), a transcription factor that plays a key role in the antiviral immune response by inducing early Interferon β (IFNβ) expression and ISG production. Additionally, IRF1 controls the expression of toll-like receptor 2 and 3 (TLR2 and TLR3) (31), both of which regulate the inflammatory response to viral infection (49, 50). The 50% reduction in the protective efficacy of vaccination against virulent VEEV was reported in an IRF1^-/-^ mouse model of the VEE disease (51), suggesting the importance of this transcription factor in host response to VEEV infection. However, how IRF1 affects VEEV infection is unclear; this might be done through the regulation of early IFN-I or inflammatory responses. We observed a significant decrease in TLR2 expression at 16 and 24 hpi in Snhg15 KD cells. Activated TLR2 signal through NF-kB and IRFs, which leads to the expression of IFN-I and proinflammatory mediators (52). Therefore, decreased expression of both IRF1 and TLR2 can alter IFN-I and proinflammatory responses to infection. Although we did not observe changes in the activation of the NF-kB signaling pathway in response to Snhg15 KD, we observed a slight decrease (from 6-fold to 2.7-fold) in IFN-β transcript levels in Snhg15 KD cells throughout the entire infection period. Notably, the regulatory effects of Snhg15 on IRF1 and TLR2 expressions have not been studied. However, our RNA-seq data suggest that Snhg15 may affect VEEV TC-83 infection by modulating the expression of these genes, therefore regulating antiviral response to infection.

Snhg15 suppression significantly reduced the mRNA levels of activating transcription factor 3 (Atf3) and proto-oncogene serine/threonine kinase (Pim1) in primary mouse astrocytes following TC-83 infection. ATF3 is a stress-induced transcription factor that modulates genes involved in different cellular processes, including inflammation and antiviral responses (30, 53, 54). ATF3 has been reported as an antiviral factor during Zika virus infection, as ATF3 depletion decreased expression of antiviral genes, such as STAT1 and IFIT1, and increased viral RNA (30). However, siRNA depletion of ATF3 decreased TC-83 infection to about 40%, suggesting ATF3 as a proviral factor during VEEV infection (55). Similarly, Pim1 acts as a proviral factor during Zika infection. Pim1 suppression/inhibition has been reported to increase STAT1 and STAT2 phosphorylation, thereby increasing ISG production and reducing Zika viral RNA expression (56). Further investigations will shed light on the regulatory impact of Snhg15 on Atf3 and Pim1, and on their functions during TC-83 infection.

In addition to regulating innate antiviral response genes, Snhg15 suppression decreased the expression of Hbegf, Ankrd33b, and H2-K2 in VEEV TC-83-infected primary mouse astrocytes. Heparin-binding EGF-like growth factor (HBEGF) has been shown to reduce influenza A virus (IAV) titers in A549 cells (57). The functions of ankyrin repeat domain 33B (Ankrd33b) and histocompatibility 2, K region locus 2 (H2-K2) are still unknown. Whether Snhg15 modulates TC-83 infection through the modulation of these genes warrants further investigation.

Our KEGG pathway analysis confirmed negative enrichment of antiviral pathways, including RIG-I-like receptor signaling, NOD-like receptor signaling, and C-type lectin receptor signaling, in response to Snhg15 suppression. Furthermore, signaling pathways that are involved in host defense against other viral infections, like pathways activated during coronavirus disease, Epstein-Barr virus infection, human T-cell leukemia virus 1, Kaposi sarcoma-associated herpes virus infection, hepatitis B virus, hepatitis C virus, human cytomegalovirus infection, and measles, were negatively enriched in response to Snhg15 suppression (Table 3).

**TABLE 3:** KEGG pathways shared at all time points after TC-83 infection in Snhg15 KD cells

Signaling pathway	Normalized enrichment score (NES)		
	8 hpi	16 hpi	24 hpi
TNF signaling pathway	−2.03	−2.91	−2.72
Ribosome biogenesis in eukaryotes	−1.54	−2.35	−2.35
NF-kappa B signaling pathway	-2	−2.34	−2.34
IL-17 signaling pathway	−1.89	−2.08	−2.33
Cytosolic DNA-sensing pathway	−1.78	−1.87	−2.15
Coronavirus disease - COVID-19	−1.45	−1.62	-2
Epstein-Barr virus infection	−1.73	−1.8	−1.96
Legionellosis	−1.51	−1.81	−1.92
Rheumatoid arthritis	−1.71	−1.65	−1.88
C-type lectin receptor signaling pathway	−1.5	−1.64	−1.87
AGE-RAGE signaling pathway in diabetic complications	−1.37	−1.94	−1.8
Chagas disease	−1.35	−1.78	−1.8
NOD-like receptor signaling pathway	−1.68	−1.73	−1.73
Human T-cell leukemia virus 1 infection	−1.5	−1.58	−1.71
Chemokine signaling pathway	−1.36	−1.65	−1.71
Kaposi sarcoma-associated herpesvirus infection	−1.56	−1.68	−1.7
Lipid and atherosclerosis	−1.48	−1.67	−1.7
Osteoclast differentiation	−1.51	−1.55	−1.62
Hepatitis B	−1.46	−1.9	−1.62
Hepatitis C	−1.48	−1.69	−1.6
Viral protein interaction with cytokine and cytokine receptor	−1.9	−1.55	−1.59
Toll-like receptor signaling pathway	−1.7	−1.81	−1.53
Human cytomegalovirus infection	−1.39	−1.44	−1.52
RIG-I-like receptor signaling pathway	−1.64	−1.6	−1.51
Acute myeloid leukemia	−1.47	−1.7	−1.43
Measles	−1.42	−1.41	−1.43
Transcriptional misregulation in cancer	−1.49	−1.42	−1.38

**Fig 8:**
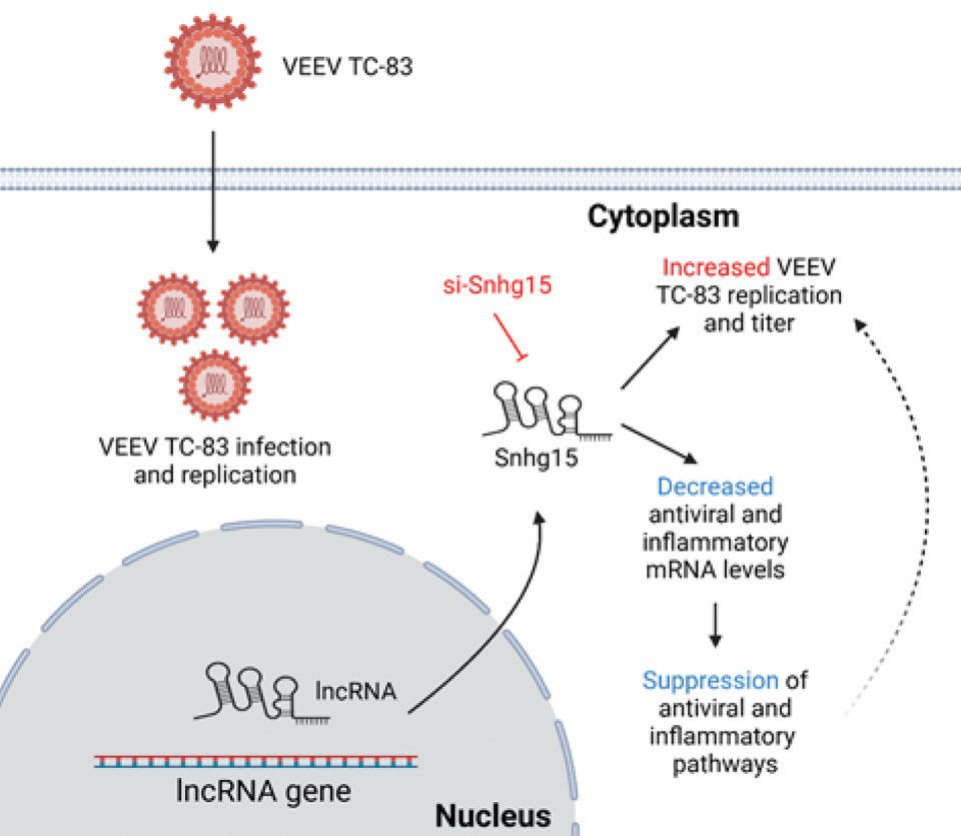
VEEV TC-83 infection induces expression of the lncRNA Snhg15, which modulates antiviral and inflammatory pathways and suppresses viral replication.

Together, our data show, for the first time, that VEEV infection of primary target cells induces different lncRNA profiles, depending on whether wild-type or attenuated VEEV is used. We identified lncRNAs that modulate TC-83 infection and highlighted Snhg15 as a potential regulator of innate antiviral pathways during TC-83 infection. Our results provide insight into how decreased Snhg15 expression may enhance TC-83 replication (Fig. 8). We showed that this lncRNA does not exert its effects on TC-83 infection by modulating the NF-kB signaling pathway. Although our study provides valuable insights into this field, we acknowledge that it also has several limitations. Additional investigations are needed to validate that significantly modulated lncRNAs during TC-83 infection are not significantly changed during TrD infection, as shown in RNA-seq. These additional experiments confirm that the observed lncRNA response was exclusive to TC-83 infection. Additionally, the lncRNA loss-of-function experiments in TrD-infected cells can shed light on the potential impact of the reported lncRNAs on TrD replication. Further investigation of changes in the protein levels of genes suppressed at the transcript level during Snhg15 KD in TC-83 infected cells will shed light on the mechanism Snhg15 uses to control VEEV infection.

## MATERIALS AND METHODS

### Cell culture

Mouse C57 brain mixed primary astrocytes (Lonza, M-ASM-330) were grown in ABM Basal Medium (Lonza, CC-3187) supplemented with AGM SingleQuots (Lonza, CC-4124). At passage five, the cells were seeded into six-well plates (5 × 10^5^ cells/well), maintained in supplemented ABM Basal Medium at 37°C and 5% CO_2_ for 24 h. Primary mouse cortical neurons (Gibco, A15586) were seeded into six-well plates (1 × 10^6^ cells/well) 4 days before infection. The plates were precoated with poly-D-lysine (25 μg/mL), and the cells were maintained in Neurobasal Medium (Gibco, 21103) supplemented with 0.5 mM GlutaMAX-I (Gibco, 35050) and 2% (vol/vol) B-27 (Gibco, 17504) at 37°C and 5% CO_2_ until infection. Vero E6 cells (ATCC, CRL-1586) were cultured in six-well plates (0.3 × 10^6^ cells/well) and maintained at 37°C and 5% CO_2_ in Dulbecco’s modified Eagle medium (DMEM) supplemented with 10% fetal bovine serum (FBS) and 1% Pen Strep Glutamine (100×) (Gibco, 10378016).

### Viruses

Wild-type VEEV TrD and the live-attenuated vaccine VEEV TC-83 strains were obtained from the Biodefense and Emerging Infections Research Resources Repository (BEI Resources, NR-332 and NR-63). Both viral stocks were expanded in Vero E6 cells and quantified using plaque assays. All TrD work was conducted in an approved select agent BSL3 facility, and RNA was inactivated prior to removal for sequencing as described below.

### RNA sequencing of VEEV-infected cells

Primary mouse astrocytes at passage five and primary mouse neurons were cultured in six-well plates as explained in the cell culture section. Infections were performed 24 h after seeding for primary mouse astrocytes (three biological replicates) and 4 days post-seeding for primary mouse neurons (three biological replicates were infected with TC-83 and two with TrD). On the day of infection, the cells were infected with either VEEV TC-83 or VEEV TrD at an MOI of 5 for 1 h. After 1 h, the viral inoculum was replaced with fresh pre-warmed cell culture medium, and the cells were incubated at 37°C and 5% CO_2_. Total RNA was extracted from uninfected cells and infected cells at 16 and 24 h post-infection using the Direct-Zol RNA Microprep Kit (Zymo Research, R2060) according to the manufacturer’s protocol. Total RNA was quantified using the Qubit RNA High Sensitivity Assay Kit (Invitrogen, Q32855) on a Qubit4, following the manufacturer’s protocol. The total RNA extracted from infected and uninfected cells was fragmented as described previously (28). Ribosomal RNA was depleted using the RiboGone Kit (Takara Bio, Cat. No. 634846) according to the manufacturer’s instructions. The RNA was prepared for sequencing using the SMARTer Universal Low Input RNA Kit for Sequencing (Takara Bio) following the manufacturer’s instructions. Samples were then prepared using the Ion Plus Fragment Library Kit (Ion Torrent) at the UNM ATG core facility, starting at the Ligation and Nick Repair step, with the only modification being the use of 80% ethanol instead of 70% ethanol for bead washes. Samples were loaded onto the Ion 540 Chip (Ion Torrent) and run on the Ion S5 XL Machine. These methods were previously published by Brown et al. and Brayer et al. (58,58). The RNA-seq reads were aligned to the mouse reference genome (MM10) using the STAR aligner. Differential gene expression analysis was performed using the DESeq2 library in R version 4.2.2. KEGG pathway analyses were conducted in R version 4.2.2 using the clusterProfiler, enrichplot, ggplot2, and Tidyverse libraries. The KEGG pathway analysis was performed using the R codes published at https://learn.gencore.bio.nyu.edu/rna-seq-analysis/gene-set-enrichment-analysis/.

### Validation of lncRNA expression

Five biological replicates of primary mouse astrocytes at passage five were cultured in six-well plates (5 × 10^5^ cells/well) and maintained in ABM Basal Medium (Lonza, CC-3187) supplemented with AGM SingleQuots (Lonza, CC-4124) at 37°C and 5% CO_2_ for 24 h. After 24 h, the cells were incubated with VEEV TC-83 (MOI 5) for 1 h or left uninfected as controls. The viral inoculum was replaced with cell culture medium 1 h after infection, and the cells were incubated at 37°C and 5% CO_2_. Infected and uninfected cells were lysed using Tri-reagent (Zymo Research, R2050-1-200) at 16 and 24 h post-infection. Total RNA was extracted from the cell lysates using the Direct-Zol RNA Microprep Kit (Zymo Research, R2060) according to the manufacturer’s protocol. The extracted RNAs were stored at −80°C for further RT-qPCR analysis.

### RT-qPCR: validation of lncRNA expression

In total, 500 ng of total RNA extracted from uninfected or VEEV TC-83 (MOI 5) infected primary mouse astrocytes at 16 and 24 h post-infection was subjected to cDNA synthesis using Superscript II reverse transcriptase (Invitrogen, 18064014) according to the manufacturer’s protocol. The amount of RNA was back-calculated in cDNA to use 25 ng of RNA/reaction. TaqMan gene expression assays, including Mm01244073_m1 (A530013C23Rik), Mm04278894_m1 (4930455J16Rik), Mm01293535_m1 (3110039M20Rik), Mm03455851_s1 (Miat), Mm01716204_m1 (Mir155hg), Mm01179930_m1 (Snhg15), Mm01319594_m1 (Mir9-3hg), ARNKU76 (GM3734), and Mm99999915_g1 (GAPDH), were purchased from Thermo Fisher Scientific to measure the expression levels of lncRNAs and GAPDH. The qPCR was performed on a QuantStudio 5 Real-Time PCR Machine (Applied Biosystems, USA), with two wells tested per sample. The expression levels of lncRNAs in each sample were measured relative to GAPDH and normalized to the expression of lncRNAs in uninfected cells using the ΔΔCt method. Five biological replicates of primary mouse astrocytes were used in this assay. Graphs were generated using GraphPad Prism (Version 10), and one-way ANOVA followed by multiple comparisons was used to assess statistical changes in the expression of lncRNAs.

### siRNA screening

Three biological replicates of mouse C57 primary mouse astrocytes (Lonza, M-ASM-330) at passage five were cultured into six-well plates (5 × 10^5^ cells/ well) and maintained in ABM Basal Medium (Lonza, CC-3187) supplemented with AGM SingleQuots (Lonza, CC-4124) at 37°C and 5% CO_2_. The siRNA SMARTpools purchased from for this assay are as follows: Lincode Mouse Mir9-3hg (101694) siRNA-SMARTpool (Horizon Discovery, R-160159-00-0005), Lincode Mouse 3110039M20Rik (67293) siRNA-SMARTpool (Horizon Discovery, R-173963-00-0005), Lincode Mouse A530013C23Rik (329562), siRNA-SMARTpool (Horizon Discovery, R-053806-00-0005), Lincode Mouse Miat (330166) siRNA-SMARTpool (Horizon Discovery, R-066744-00-0005), and Lincode Non-Targeting Pool (Horizon Discovery, D-001320-10-20). The sequence of the siRNA used to suppress Snhg15 can be found in a previous study (33). Twenty-four hours post-seeding, the cells were transfected with 50 nM of siRNA targeting lncRNA or a pool of nontargeting siRNAs (control) using TransIT-X2 transfection reagent (Mirus, MIR6004) using the manufacturer’s protocol, with the only modification being the use of half of the recommended transfection reagent/rxn. Each siRNA pool was transfected to two wells, except for the siRNA used against Snhg15 that was transfected into one well. Twenty-four hours after transfection, the cells were infected with VEEV TC-83 (MOI 5) for 1 h or left uninfected (control), with the exception of cells transfected with siRNA against Snhg15 that were infected with TC-83 at MOI 0.5. The viral inoculum was replaced with cell culture medium after 1 h, and the cells were incubated at 37°C and 5% CO_2_ for 24 h. At 24 h post-infection, the cells were lysed using Tri-reagent (Zymo Research, R2050-1-200), and the total RNA was extracted from the cell lysates using Direct-Zol RNA Microprep Kit (Zymo Research, R2060) according to the manufacturer’s protocol. The total RNAs were stored at −80°C for later RT-qPCR analysis.

### RT-qPCR: validation of lncRNA suppression

To measure the expression levels of lncRNA after lncRNA suppression, total RNA was extracted from cells under the following conditions: (i) treated with a pool of four nontargeting siRNAs as a control for baseline lncRNA expression; (ii) transfected with siRNA targeting the lncRNA followed by infection with VEEV TC-83 (MOI 5 or 0.5); and (iii) transfected with a pool of four nontargeting siRNAs followed by infection with VEEV TC-83 (MOI 5 or 0.5). Total RNA from these samples was subjected to cDNA synthesis using Superscript II reverse transcriptase (Invitrogen, 18064014) according to the manufacturer’s protocol. The volume of cDNA containing 25 ng of total RNA was used in each reaction. TaqMan gene expression assays, Mm01244073_m1(A530013C23Rik), Mm01293535_m1(3110039M20Rik), Mm01179930_m1 (Snhg15), Mm01319594_m1 (Mir9-3hg), Mm03455851_s1 (Miat), and Mm99999915_g1 (GAPDH) were purchased from Thermo Fisher Scientific to measure the expression levels of lncRNAs and GAPDH. The qPCR was performed in a QuantStudio 5 Realtime PCR Machine (Applied Biosystems, USA). The expression of each lncRNA was tested in four qPCR wells, with the exception of Snhg15 expression, which was tested in three wells. The expression level of lncRNA in each sample was calculated relative to GAPDH and normalized to the expression of lncRNA in primary mouse astrocytes transfected with a pool of non-targeting siRNA using the ΔΔCt method. Three biological replicates of primary mouse astrocytes were used in this assay.

### Plaque assays

To measure the potential effect of lncRNA suppression on the viral titer, Vero E6 cells were seeded into six-well plates (0.5 × 10^6^ cells/well) 24 h prior to infection. Twenty-four hours post-seeding, the cells were incubated with 10-fold serial dilutions of supernatants from lncRNA-suppressed primary mouse astrocytes that are infected with VEEV TC-83 (MOI 5), primary mouse astrocytes transfected with a pool of four non-targeting siRNAs (negative control), or primary mouse astrocytes transfected with a pool of four non-targeting siRNAs followed by infection with VEEV TC-83 (MOI 5) (positive control) for 1 h at 37°C with rocking at 30 min. The supernatant used for plaque assay was collected at 24 hpi. Dilutions 10^−1^ through 10^−9^ of the supernatants were used in the plaque assay. After the incubation period, the inoculum was replaced with 1 mL of 1% agarose in modified Eagle medium supplemented with 2.5% FBS. The plates were incubated at 37°C and 5% CO_2_ for 48 h. To fix the cells, 1 mL of 4% formaldehyde was added to the agar overlay, and the plates were incubated at 4°C overnight. After the incubation period, the agar overlay was removed, and the fixed monolayer was stained with 0.8% crystal violet solution. Plaques were quantified, and the virus concentrations were recorded as PFU/mL. Three wells per dilution were tested. Three biological replicates were used in this assay.

### RNA sequencing of Snhg15 knockdown cells

Three biological replicates of primary mouse astrocytes (Lonza, M-ASM-330) at passage five were cultured into six-well plates and maintained in astrocyte growth medium at 37°C and 5% CO_2_ for 24 h. At 24-h post-seeding, the cells were transfected with either siRNA targeting Snhg15 or a pool of four non-targeting siRNAs (Control) using TransIT-X2 transfection reagent (Mirus, MIR6004) according to the manufacturer’s protocol with modification of using half of the suggested transfection reagent. The transfected cells were incubated at 37°C and 5% CO_2_ for 24 h; 24 h after transfection, the cells were incubated with VEEV TC-83 (MOI 5) for 1 h with rocking at 30 min. After 1 h, the viral inoculum was replaced with cell culture medium, and cells were incubated at 37°C and 5% CO_2_ for another 24 h. At 24 h post-infection, the cells were lysed using Tri-reagent (Zymo Research, R2050-1-200), and total RNA was extracted from the cell lysates using Direct-Zol RNA Microprep Kit (Zymo Research, R2060) according to the manufacturer’s protocol. Total RNA samples were sent to the University of Colorado-Anschutz sequencing center for RNA sequencing. After the ribosomal RNA depletion, the RNA sequencing was run on the NovaSeqX Machine. The paired-end sequencing was done with the read depth of 50 million per sample. The obtained reads were aligned to the mouse reference genome (MM10) using STAR aligner, and differential gene expression analysis was done in R version 4.2.2 using the DESeq2 library. KEGG pathway analyses were performed in R version 4.2.2 using clusterProfiler, enrichplot, ggplot2, and tidyverse libraries and using R codes published at https://learn.gencore.bio.nyu.edu/rna-seq-analysis/gene-set-enrichment-analysis/. The Venn diagram was generated in R using version 4.2.2 and using the VennDiagram and grid libraries.

### Fluorescent microscopy

To investigate whether Snhg15 suppression will modulate the NF-kB nuclear localization, we performed fluorescent microscopy. Primary mouse astrocytes at passage five were seeded into a cell culture/imaging microplate (Agilent, 204626-100) at a density of 1.2 × 10^4^ cells per well and maintained in ABM Basal Medium (Lonza, CC-3187) supplemented with AGM SingleQuots (Lonza, CC-4124) at 37°C and 5% CO_2_. At 24 h after seeding, the cells were transfected with 50 nM siRNA against Snhg15 (33) or a pool of four non-targeting siRNAs (Dharmacon, D001320-10-20) using TransIT-X2 transfection reagent (Mirus, MIR6004) using the manufacturer’s protocol, with the only modification being the use of half of the recommended transfection reagent/rxn. At 24 h after transfection, the cells were incubated with a TC-83 (MOI 5) inoculum for 1 h. After the incubation period, the cell culture medium was changed to fresh medium. One group of cells was transfected with a pool of four non-targeting siRNAs (Dharmacon, D-001320-10-20) and left uninfected as a control for changes in NF-kB(p65) nuclear localization following transfection. At 24 h after infection, the cells were fixed with 4% paraformaldehyde (Thermo Fisher Scientific, J19943-K2) for 20 min at room temperature, followed by permeabilization with 0.05% Triton X-100 for 15 min at room temperature. After the permeabilization period, the cells were blocked in 10% normal goat serum (Vector Laboratories, S-1000-20) in 1× PBS for 1 h and then incubated with Anti-NF-kB p65 antibody [E379] (Abcam, ab32536) diluted in 10% normal goat serum in 1× PBS at the concentration of 1 µg/mL for 2 h at room temperature on a rocker. The cells were then washed with 1× PBS and incubated with Goat Anti-Rabbit IgG Fc (Alexa Fluor 647) (Abcam, ab150091) diluted in 10% normal goat serum in 1× PBS at a concentration of 2 µg/mL for 1 h at room temperature, in the dark and on a rocker. After the incubation period, the cells were washed and incubated with Hoechst 33342 (Thermofisher Scientific, H3570) diluted in 1× PBS at a concentration of 5 µg/mL for 30 min at room temperature and in the dark. Cells were washed and incubated with HCS Cell Mask Green Stain (Thermo Fisher Scientific, H32714) at a concentration of 2 µg/mL for 30 min in the dark. The cells were washed with 1× PBS, and images were captured on Cellinsight CX7 High-content Analysis platform (ThermoFisher Scientific) at 20× (0.7 NA) Air objective magnification using HCS software. Fields were acquired until 2,000 valid cells could be analyzed/well. Raw images were analyzed with Colocalization BioApplication in HSC Studio. Individual cells were segmented according to channel 1, HCS Cell Mask Green Stain (Thermo Fisher Scientific, H32714), the nuclear region of interest was defined by Hoechst channel 2, and p65 was defined by channel 3. Analysis used from ROI_A_Target_I_ObjectTotalIntensity. Images were chosen as representative of the mean for the fields, and a single cell represents the mean total intensity of p65 in the nucleus. The graph was generated using GraphPad Prism version 10, and one-way ANOVA with multiple comparisons was used to measure statistical significance.

### Western blot

We measured the effect of Snhg15 suppression on NF-kB signaling pathway activation by assessing changes in p65 protein levels and the phosphorylation levels of p65 using western blot. Briefly, primary mouse astrocytes were cultured in six-well plates as described previously in the cell culture section. At 24 h after seeding, the cells were transfected with 50 nM siRNA targeting Snhg15 (33) or a pool of four non-targeting siRNAs using TransIT-X2 (Mirus, Mir6000) according to the manufacturer’s protocol, with the exception of using half of the transfection reagent recommended by the manufacturer. At 24 h after transfection, both groups of cells were incubated with TC-83 inoculum (MOI 5) for 1 h. After an incubation period, the viral inoculum was replaced with ABM Basal Medium (Lonza, CC-3187) supplemented with AGM SingleQuots (Lonza, CC-4124), and cells were maintained at 37°C and 5% CO_2_. Cells transfected with a pool of four non-targeting siRNA were used as a control for basal p65 phosphorylation levels. At 24 hpi, proteins were extracted using RIPA lysis and extraction buffer (Thermo Fisher Scientific, 89900) supplemented with Halt protease and phosphatase inhibitor single-use cocktail (Thermo Fisher Scientific, 78442). Total protein levels were quantified using the Qubit protein assay kit (Invitrogen, Q33211) according to the manufacturer’s protocols. Equal concentration of proteins loaded into Bolt 4%–12% Bis-Tris Plus WedgeWell Gel (Invitrogen, NW04122BOX) and transferred to PVDF membrane using an iBlot 2 Gel Transfer device (Invitrogen). The membranes were blocked in 1× Pierce Clear Milk Blocking Buffer (Thermo Fisher Scientific, ZB4139391) in 1× PBS for 1 h at room temperature on a rocker. The blocking buffer was then replaced with a cocktail of primary antibodies diluted 1:1,000 in 1× blocking buffer, including NF-κB p65 (D14E12) XP Rabbit mAb (Cell signaling, #8242) or Phospho-NF-κB p65 (Ser536) (93H1) Rabbit mAb (Cell signaling, #3033), with β-Actin (8H10D10) Mouse mAb (Cell signaling, #3700), overnight at 4°C. After an incubation period, the cells were incubated with a cocktail of secondary antibodies diluted 1:10,000 in 1× blocking buffer, including goat anti-rabbit IgG and donkey anti-mouse IgG antibodies, to target p65 and p-p65, and β-actin for 1 h at room temperature on the Rocker. The blots were imaged on Odyssey Imager (Licorebio). The images were processed by ImageJ for better visualization.

### Enzyme-linked immunosorbent assay (ELISA)

Primary mouse astrocytes were cultured in six-well plates as defined previously in the cell culture section. At 24 h after seeding, the cells were transfected with 50 nM of siRNA targeting Snhg15 or a pool of four nontargeting siRNAs using TransIT-X2 (Mirus, Mir6000) with the only modification being half of the recommended transfection reagent used. For each transfection, one group of transfected cells was incubated with TC-83 (MOI 5) for 1 h, while the other group remained uninfected. Additionally, non-transfected cells were incubated with TC-83 (MOI 5) for 1 h or left uninfected. For consistency, the medium from both infected and uninfected transfected and non-transfected cells was replaced with fresh ABM Basal Medium (Lonza, CC-3187) supplemented with AGM SingleQuots (Lonza, CC-4124) at 1 h post-infection, and the cells were maintained at 37°C and 5% CO_2_. One group of untreated cells was treated with 10 ng/mL recombinant rat TNF-alpha protein (R&D Systems, 510-RT-010) for 24 h as a control for proinflammatory mediator production and release. The supernatant from all groups was collected at 24 h post-infection or post-treatment and used for ELISA assays. The ELISA assays were done using Mouse IL-6 ELISA Kit (RayBiotech, ELM-IL6-1), Mouse KC ELISA Kit (RayBiotech, ELM-KC-1), MIP-2 ELISA Kit (RayBiotech, ELM-MIP2-1), and Mouse RANTES ELISA Kit (RayBiotech, ELM-RANTES-1) according to the manufacturer’s protocols. The graphs were generated using GraphPad Prism Version 10, and one-way ANOVA followed by multiple comparisons was used to measure statistical significance.

See Table 4 for more information on methods used in the study.

**TABLE 4:** STAR methods

Reagent or resource	Source	Identifier
Virus strains		
VEEV Trinidad Donkey (VEEV IA/B)	BEI Resources	NR-332
VEEV TC-83 (Vaccine strain)	BEI Resources	NR-63
Experimental models: cells		
Mouse C57 brain mixed primary astrocytes	Lonza	M-ASM-330
Primary mouse cortical neurons	Gibco	A15586
Vero E6 cells	ATCC	CRL-1586
TaqMan gene expression assays		
A530013C23Rik	Thermo Fisher Scientific	Mm01244073_m1
4930455J16Rik	Thermo Fisher Scientific	Mm04278894_m1
3110039M20Rik	Thermo Fisher Scientific	Mm01293535_m1
Miat	Thermo Fisher Scientific	Mm03455851_s1
Mir155hg	Thermo Fisher Scientific	Mm01716204_m1
Snhg15	Thermo Fisher Scientific	Mm01179930_m1
Mir9-3hg	Thermo Fisher Scientific	Mm01319594_m1
GM3734	Thermo Fisher Scientific	ARNKU76
GAPDH	Thermo Fisher Scientific	Mm99999915_g1
VEEV TC-83	Sequence published in reference 28	
siRNAs		
Lincode mouse Mir9-3hg(101694)- siRNA SMARTpool	Horizon Discovery	R-160159-00-0005
Lincode mouse 3110039M20Rik (67293)siRNA-SMARTpool	Horizon Discovery	R-173963-00-0005
Lincode mouse A530013C23Rik (329562) siRNA-SMARTpool	Horizon Discovery	R-053806-00-0005
Lincode mouse miat(330166) siRNA-SMART pool	Horizon Discovery	R-066744-00-0005
Lincode non-targeting pool −20 nM	Horizon Discovery	D-001320-10-20
si-Snhg15	Sequence published in reference 33	
Plasmid		
TC-83 plasmid	Kindly provided by Dr. Kyleen Khen-Hall	
Reagents and commercial kits		
ABM basal medium	Lonza	CC-3187
AGM SingleQuots	Lonza	CC-4124
Neurobasal medium	Gibco	21103
GlutaMAX-I	Gibco	35050
B-27	Gibco	17504
TransIT-X2 transfection reagent	Mirus	MIR6004
4% Paraformaldehyde	Thermo Fisher Scientific	J19943-K2
Goat serum	Vector Laboratories	S-1000-20
RIPA lysis and extraction buffer	Thermo Fisher Scientific	89900
Halt protease and phosphatase inhibitor single-use cocktail	Thermo Fisher Scientific	78442
Bolt 4%–12% Bis-Tris Plus WedgeWell Gel	Invitrogen	NW04122BOX
1× Pierce clear milk blocking buffer	Thermo Fisher Scientific	ZB4139391
Tri-reagent	Zymo Research	R2050-1-200
Superscript II reverse transcriptase	Invitrogen	18064014
Direct-Zol RNA microprep kit	Zymo Research	R2060
Qubit RNA high sensitivity assay Kit	Invitrogen	Q32855
RiboGone kit	Takara Bio	634846
Mouse IL-6 ELISA kit	RayBiotech	ELM-IL6-1
Mouse KC ELISA kit	RayBiotech	ELM-KC-1
Mouse MIP-2 ELISA kit	RayBiotech	ELM-MIP2-1
Mouse RANTES ELISA kit	RayBiotech	ELM-RANTES-1
Qubit protein assay kit	Invitrogen	Q33211
Antibodies and cell stains		
Anti-NF-kB p65 antibody (E379)	Abcam	ab32536
Goat anti-rabbit IgG Fc (Alexa Fluor 647)	Abcam	ab150091
Hoechst 33342	Thermo Fisher Scientific	H3570
HCS CellMask Green Stain	Thermo Fisher Scientific	H32714
NF-κB p65 (D14E12) XP rabbit mAb	Cell Signaling	#8242
Phospho-NF-κB p65 (Ser536) (93H1) rabbit mAb	Cell Signaling	#3033
β-Actin (8H10D10) mouse mAb	Cell Signaling	#3700
Software		
R version 4.2.2	https://cran.rstudio.com/	
GraphPad Prism	https://www.graphpad.com/features	

## Data Availability

The RNA sequencing data are available in GEO under accession numbers GSE324479, GSM9578214–GSM9578249, and GSE324524, GSM9579016–GSM9579054. This research did not generate novel reagents. Additional information required for reanalyzing the data reported in this paper is available upon request to the lead contact, Dr. Steven B. Bradfute.

## SUPPLEMENTAL MATERIAL

Supplemental figures jvi.01353-25-s0001.docx Figures S1 to S7.

ASM does not own the copyrights to Supplemental Material that may be linked to, or accessed through, an article. The authors have granted ASM a non-exclusive, world-wide license to publish the Supplemental Material files. Please contact the corresponding author directly for reuse.
