# Supplementary material for: Identification of host lncRNAs that impact Venezuelan equine encephalitis virus TC-83 replication
Source: J Virol. 2026 Apr 23;100(5):e01353-25. doi: 10.1128/jvi.01353-25 (PMC13185544; doi:10.1128/jvi.01353-25)
Supplement: Supplemental figures — Figures S1 to S7. [file jvi.01353-25-s0001.docx]

**Supplementary Figures**

**C**

**Supplementary Figure 1. Mapped reads confirmed successful VEEV infection and replication in primary mouse astrocytes and neurons.** Barplots show reads mapped to mouse and VEEV genome in primary mouse astrocytes **(A)** and primary mouse neurons **(B)**, infected with either VEEV TC-83 (Left) or VEEV TrD (Right) at 16h and 24h post-infection. uninfected cells used as control for these experiments. **(C),** more rapid replication of TrD compared to TC-83 was observed in supernatants of infected astrocytes.

**A**

**B**

**Supplementary Figure 2. The host cellular response to VEEV varies depending on the VEEV strain and cell type.** Volcano plots show DEG in primary mouse astrocytes **(A)**, primary mouse neurons **(B)**, infected with either VEEV TC-83 (Left) or VEEV TrD (Right) for 24hours. p-value threshold adjusted to p.Adj value = 0.05

**S. Figure 2. The host cellular response to VEEV varies depending on the VEEV strain and cell type.** Volcano plots show DEG in primary mouse astrocytes **(A),** primary mouse neurons **(B)**, and primary mouse DCs **(C**), infected with eighter VEEV TC-83 (Left) or VEEV TrD (Right) for 24hours. p-value threshold adjusted to p. Adjusted value = 0.05

**Supplementary Figure 3. KEGG pathway analyses confirmed the activation of a greater number of antiviral signaling pathways in TC-83-infected astrocytes compared to TrD-infected cells.**

**Supplementary Figure 4. KEGG pathway analyses confirmed the activation of a greater number of antiviral signaling pathways in TC-83-infected neurons compared to TrD-infected cells.**

**A B**

**C D**

**Supplementary Figure 5.** **The host cellular lncRNA response to VEEV strains at 16hpi. A-B)** Volcano plots show DE-lncRNAs in primary mouse astrocytes **(A)** and primary mouse neurons **(B)** infected with VEEV TC-83. **C-D)** Volcano plots show DE-lncRNAs in primary mouse astrocytes **(C)** and primary mouse neurons **(D)** infected with VEEV TrD. All the plots show the results from RNA-seq at 16h.p.i. The p-value threshold adjusted to show p.Adj value = 0.05.

**A B**


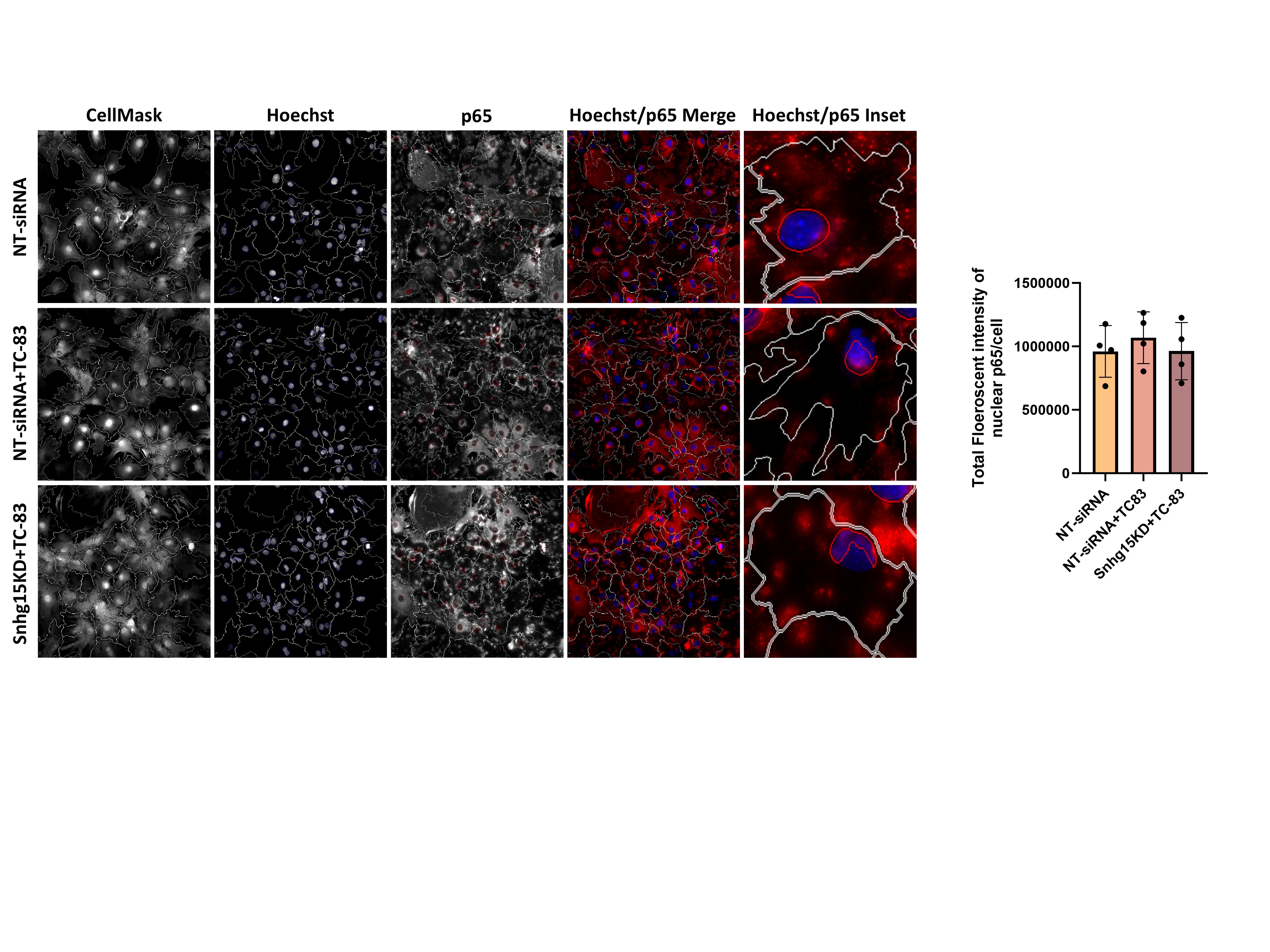


**Supplementary Figure 6: Snhg15 suppression did not alter NF-kB (p65) nuclear localization significantly in TC-83-infected primary mouse astrocytes. A)** Primary mouse astrocytes were transfected with NT-siRNA or siRNA targeting Snhg15, then infected with TC-83 (MOI 5) at 24 hours post-transfection or left uninfected. At 24 hours post-infection, cells were fixed, permeabilized, and stained with an anti-mouse NF-kB (p65) antibody (red) to measure changes in NF-kB nuclear localization in response to Snhg15 suppression. Nuclei were counterstained with Hoechst (blue), and the entire cell was stained with HCS Cell Mask green stain. Panels from left to right: Channel 1 (cell mask for single-cell segmentation - white outline), Channel 2 (Hoechst for nuclear ROI – blue outline), Channel 3 (NF-kB cytoplasmic and nuclear distribution - red outline indicating nuclear p65), Channel 4 (merged Hoechst and p65 channels), and inset (white outline for cell border, red outline for nuclear p65). All panels represent the field with the mean total nuclear p65 expression for each treatment group. Each inset shows a single cell representing the average nuclear p65 expression under each condition. All images were captured at 40x Air objective NA 0.7, with fields acquired until at least 2000 valid cells could be analyzed. **B)** Measurement of total fluorescent intensity of nuclear p65/cell. The total fluorescent intensity of nuclear p65 was measured in 2000 cells per well. Each dot represents one well per treatment. One-way ANOVA followed by multiple comparisons was used to assess statistical significance.

**A**


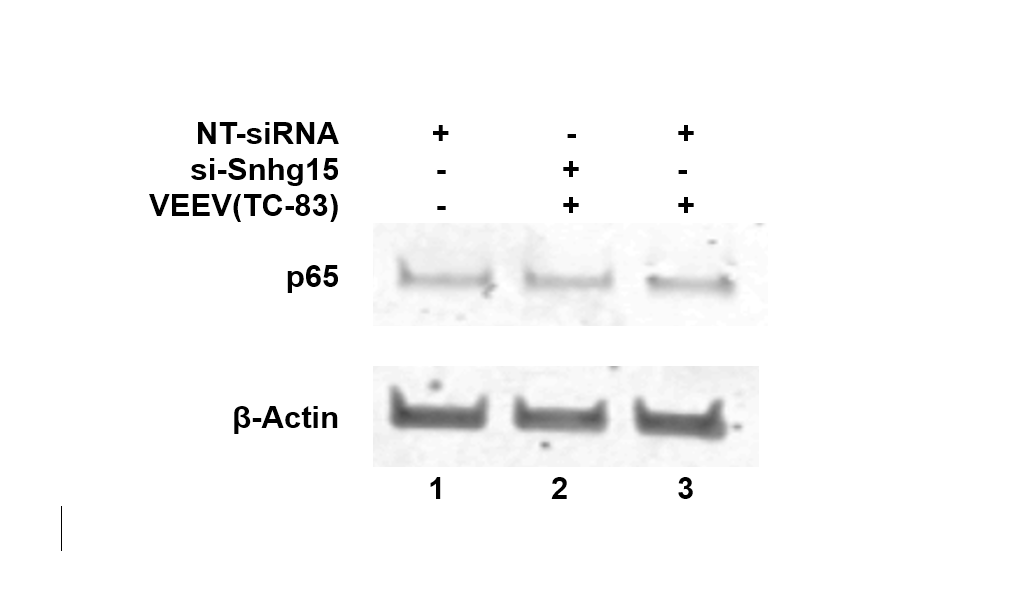

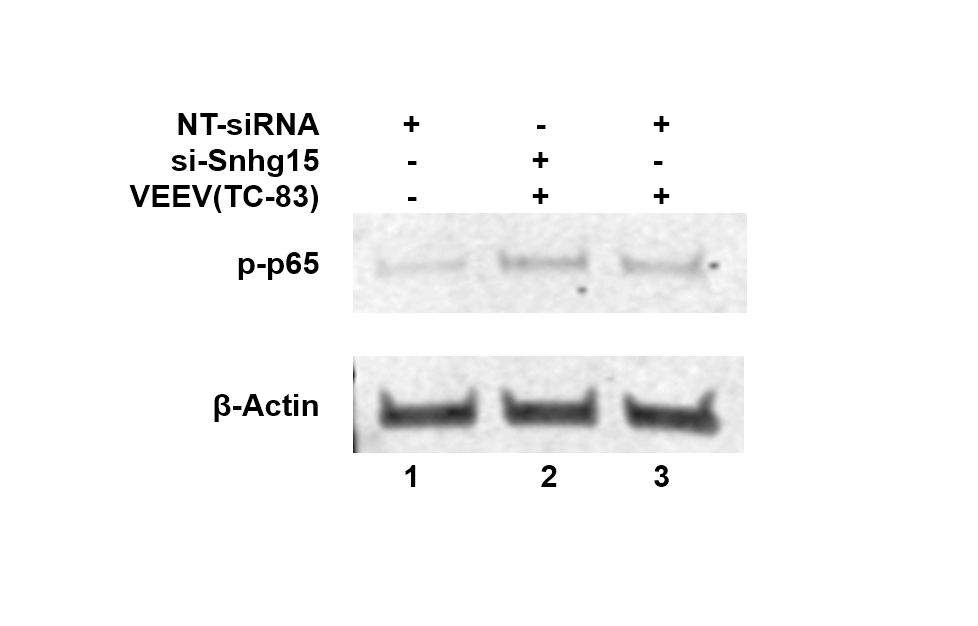


**B**

**Supplementary Figure 7. Snhg15 suppression did not alter NF-kB signaling and downstream protein production.** **A)** Primary mouse astrocytes were transfected with NT-siRNA or si-Snhg15, followed by infection with TC-83 for 24 hours. The cells transfected with NT-siRNA were used as the control for basal protein levels of p65 and p-p65, in these cells after transfection. Cells were lysed at 24hp.i. and cell lysates were subjected to Western Blot. The images were processed in ImageJ to improve the visualization of the bands. **B)** Primary mouse astrocytes were transfected with NT-siRNA or si-Snhg15 or left untreated. At 24h post-transfection, one group of NT-siRNA-treated, si-Snhg15-treated, and untreated cells was infected with TC-83 (MOI 5), and the other group was left uninfected (controls). Primary mouse astrocytes were treated with 10ng/ml recombinant rat TNF-alpha protein for 24h to use as a positive control for NF-kB activation. At 24h post-infection/treatment, supernatant from all cell groups was collected and subjected to Enzyme-Linked Immunosorbent Assay to measure changes in IL6, CXCL1, CXCL2, and CCL5 protein levels in all treatment groups. Each dot represents one well per treatment. One-way ANOVA followed by multiple comparisons was used to assess statistical significance.
